# Comparative analysis of respiratory infection and clinical outcomes in Saudi hematology: malignant versus non-malignant cohorts

**DOI:** 10.3389/fcimb.2026.1916017

**Published:** 2026-10-28

**Authors:** Zinab O. Doha, Aiah M. Khateb, Ahmed S. Barefah, Osman Radhwi, Ali Algiraigri, Lina Alhejaili, Sadeem Alhelali, Hadeel Alhazmi, Khloud Almgthwi, Esam I. Azhar

**Affiliations:** 1 Department of Clinical Laboratory Sciences, Collage of Applied Medical Science, Taibah University, Madina, Saudi Arabia; 2 Health and Life Research Center, Taibah University, Madinah, Saudi Arabia; 3 Hematology Department, Faculty of Medicine, King Abdulaziz University, Jeddah, Saudi Arabia; 4 Hematology Research Unit, King Fahd Medical Research Centre, King Abdulaziz University, Jeddah, Saudi Arabia; 5 Special Infectious Agents Unit, King Fahd Medical Research Center, King Abdulaziz University, Jeddah, Saudi Arabia; 6 Medical Laboratory Sciences Department, Faculty of Applied Medical Sciences, King Abdulaziz University, Jeddah, Saudi Arabia

**Keywords:** hematologic malignancies, opportunistic pathogens, respiratory infections, seasonal trends, survival analysis

## Abstract

**Background:**

Respiratory infections are a primary cause of mortality in malignancy, yet the temporal dynamics and pathogen profile of the respiratory tract (RT) in these patients remain poorly defined. This 13-year study characterizes the culture isolate spectrum, seasonality, and survival impacts of RT infections in malignant versus non-malignant populations.

**Methods:**

We conducted a 13-year retrospective observational study (2007–2020) at King Abdulaziz University Hospital, Jeddah, Saudi Arabia, enrolling 104 hematological patients with confirmed respiratory infections — 77 with malignant hematological disorders and 27 with non-malignant hematological disorders. Respiratory specimens were processed by conventional culture. Microbial prevalence was compared at the patient level using Fisher’s exact test with Benjamini-Hochberg FDR correction. Survival analysis was performed using Kaplan-Meier estimation with log-rank testing, stratified by infection type and pathogenicity classification. Spearman rank correlation matrices were computed to explore microbial co-occurrence patterns.

**Results:**

Malignant patients demonstrated significantly higher case-fatality rate and markedly shorter overall survival. Mixed bacterial growth was the strongest discriminator between groups (p<0.001). Opportunistic pathogens were enriched in malignant patients while strict pathogens predominated in non-malignant individuals. Spearman correlation analysis revealed diffuse co-occurrence patterns in malignant patients — consistent with immunosuppression-driven ecological instability — contrasting with tightly structured-associated clustering in non-malignant patients. Infection peaks occurred in spring and summer for malignant patients versus winter for non-malignant patients.

**Conclusion:**

Malignancy fundamentally shifts the RT environment toward ecological instability and opportunistic overgrowth. These findings underscore the necessity for microbial distribution and seasonally tailored prophylactic strategies to manage infections in immunocompromised hematological populations effectively.

## Highlights

Respiratory infections are a leading cause of death for patients with cancer, yet we still do not fully understand how these infections behave over time or how they disrupt the natural balance of microbes in the airway. This study aimed to compare the types of germs found in the respiratory tracts of cancer patients versus those without cancer over a 13-year period. Our research found that cancer patients suffer from more frequent and severe infections, often caused by multiple types of opportunistic germs that thrive when the immune system is weak. Furthermore, we discovered that infection patterns in cancer patients peak during different seasons compared to the public. These findings are important because they suggest that doctors should use specific monitoring and seasonal prevention strategies to better protect vulnerable patients, ultimately improving survival rates and clinical care.

## 1 Introduction

In the clinical landscape of the Kingdom of Saudi Arabia, respiratory tract infections (RTIs) represent a persistent challenge to public health, further complicated by the country’s unique arid climate, periodic dust storms, new viral strains, and the annual influx of millions of pilgrims during the Hajj and Umrah (Khateb et al., 2025; Salmanton-García et al., 2025). For the non-malignant population, respiratory tract (RT) pathogens are frequently transient and self-limiting. However, for patients with hematological malignancies and other cancers, these same pathogens can be tragic. Hematology patients are broadly categorized into those with malignant conditions, such as leukemia and lymphoma, and those with non-malignant disorders, such as aplastic anemia, iron deficiency or hemoglobinopathies. While both groups are susceptible to infections, their microbial dynamics differ profoundly (Bourdiol and Roquilly, 2022; Khateb et al., 2025). The interplay between host immunity, environmental stressors, and microbial virulence creates a complex dynamic that remains under-characterized in the Middle Eastern context, particularly regarding long-term seasonal trends and prognostic outcomes.

The respiratory tract serves as a primary reservoir for a diverse range of microorganisms, which can be broadly categorized into three distinct ecological groups: strict pathogens (e.g., *Influenza*, *MRSA*, *M. catarrhalis*), which consistently cause disease; opportunistic pathogens (e.g., *A. baumannii*, *Entrococcus*, *P. aeruginosa*), which exploit the weakened defenses of the immunocompromised; and commensal microbial groups, which typically maintain a symbiotic or neutral relationship with the host but may contribute to altered microbial balance in the setting of intensive chemotherapy (Murray et al., 2020). In oncology patients, the traditional boundaries between these groups often blur (Murray et al., 2020). Treatment-induced neutropenia and mucosal barrier injury (mucositis) facilitate the transition of commensals into invasive pathogens, leading to severe complications that are rarely observed in the healthy or non-malignant population.

While short-term epidemiological snapshots are available, there is a critical need for longitudinal data to understand how these microbial dynamics shift over time. Factors such as the emergence of novel viral strains, changes in regional antimicrobial resistance (AMR) patterns, and local seasonal variations significantly influence the “spectrum” of detectable RT pathogens. The respiratory pathogens shows seasonal fluctuations, which may affect infection rates and treatment responses in cancer patients, highlighting the need for tailored monitoring (Jiang and Zhang, 2025). Furthermore, the prognostic impact of these infections ranging from minor treatment delays to mortality lower respiratory tract progression varies significantly between patients undergoing active cancer therapy and the non-malignant population. Disturbances in mucosal barrier integrity and microbial balance can worsen with hematologic therapies such as chemotherapy and immunosuppression, po- tentially leading to disease progression and increased susceptibility to opportunistic in- fections. Recent advances highlight the potential of pathogen-targeted and host-directed therapies to complement existing treatments, particularly in managing graft-versus-host disease (GvHD) in stem cell transplant recipients In non-malignant conditions, a balanced mucosal flora supports hematopoiesis and immune function, which is crucial for prevent- ing conditions like iron deficiency anemia and autoimmune cytopenias (Biennier et al., 2024; Yue et al., 2024). Maintaining a healthy microbial balance is essential to prevent respiratory infection in non-malignant hematologic disorders through mechanisms like epithelial barrier disruption and antigen sequestration (Biennier et al., 2024).

This study aims to address these gaps by analyzing a dataset spanning a 13-year period (2007–2020). By investigating and comparing the microbial spectrum, seasonal dynamics, and prognostic implications of RT pathogens in cancer patients versus the healthy or non-malignant population, we seek to define the specific risks associated with strict, opportunistic, and commensal groups. These findings will provide Saudi clinicians with an evidence-based framework for improving early screening, refining empirical therapy, and optimizing infection control strategies in one of the world’s most vulnerable patient cohorts.

## 2 Materials and methods

### 2.1 Study design and setting

This retrospective observational study evaluated the epidemiology and clinical impact of respiratory tract (RT) pathogens over a 13-year period from January 2007 to December 2020 among patients with hematological disorders at King Abdulaziz University Hospital, Jeddah, Saudi Arabia. Data were extracted from microbiology and clinical records. Patients were classified into those with malignant hematologic conditions (including leukemia, lymphoma, and other neoplasms) and those with non-malignant disorders (such as aplastic anemia, iron deficiency anemia, and hemoglobinopathies). The dataset included hematologic diagnosis, dates of hospital admission, RT culture results, isolated microbial species, and survival outcomes.

Each infection episode was defined as a discrete instance of RT culture positivity accompanied by relevant clinical documentation, such as respiratory symptoms, febrile episodes, or physician-initiated new antimicrobial treatment. To distinguish infection from asymptomatic colonization, culture findings were interpreted in conjunction with clinical indicators recorded at the time of culture. While the RT is known to harbor commensal and colonizing organisms—especially in immunocompromised hosts—only episodes with evidence suggestive of active infection were retained for analysis. This definition aimed to reduce misclassification of colonization as infection while acknowledging that in hematologic malignancy patients, colonization may still pose clinical risk and precede lower respiratory tract involvement.

Data on specific cancer treatment regimens, chemotherapy exposure, comorbidities (e.g., diabetes), and detailed clinical histories were not available. The study focused on comparing microbial prevalence, seasonal trends, and survival outcomes between patients with hematological malignancies and a non-malignant hematological cohort.

### 2.2 Study population, inclusion, and exclusion criteria

Patients were eligible for inclusion if they had a documented laboratory-confirmed RT infection with an identifiable microbial organism and a clearly defined diagnosis. Only patients with complete records for year of infection, microbiological findings, diagnosis group, and mortality outcome were included. Patients were excluded if microbiological identification was absent, clinical records were incomplete, or if the infection was unrelated to the respiratory tract. A total of 179 unique patients with 506 respiratory infection episodes were screened at King Abdulaziz Hospital, Jeddah, Saudi Arabia between January 2007 and December 2020. Following exclusion of 70 patients with non-hematological diagnoses, 109 hematological patients (285 episodes) remained. A further 5 patients were excluded due to incomplete clinical metadata, yielding a final analytic cohort of 104 patients (276 episodes). Patients were classified into two groups: malignant hematological disorders (n=77, 218 episodes) and non-malignant hematological disorders (n=27, 58 episodes). The complete patient selection pathway is illustrated in **Figure 1A**.

**Figure 1:**
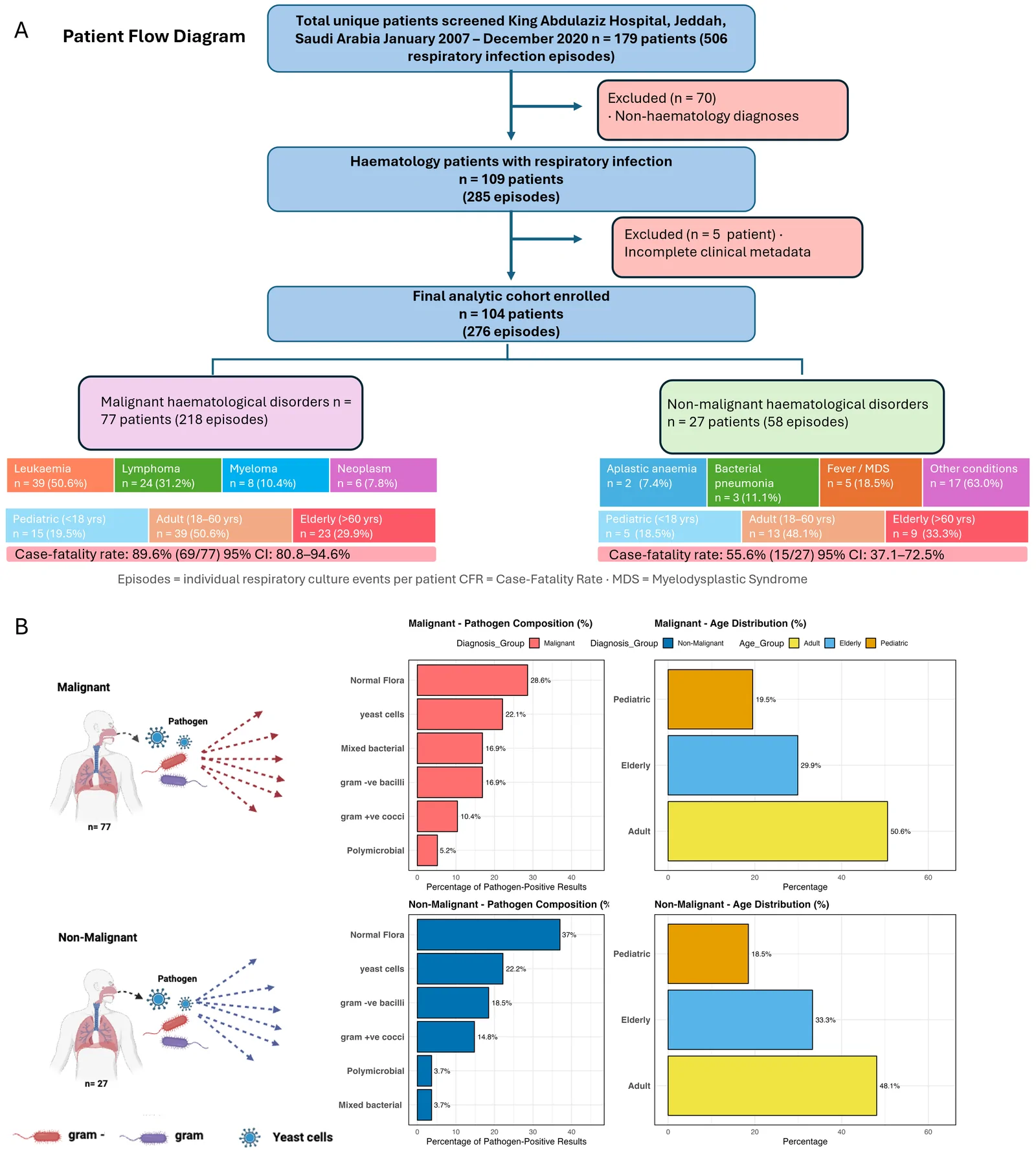
Bacterial pathogen composition among malignant and non-malignant cases of respiratory infections. **(A)** Patient enrolment flow diagram showing the selection pathway from 179 screened patients to the final analytic cohort of 104 patients (77 malignant, 27 non-malignant). **(B)** compares the distribution of microbial groups detected in respiratory specimens from patients with malignant (n = 77) and non-malignant (n = 27) diagnoses. Left panel: Schematic illustrations depict host-pathogen interaction in each clinical group. Right panel: Bar graphs show the percentage of pathogen-positive results for each microbial group, ordered by prevalence. Legend (bottom right) illustrates representative pathogen types by morphology and stain.

### 2.3 Microbiological data and pathogen classification

Respiratory specimens submitted for microbiological culture included sputum samples, throat swabs, nasopharyngeal swabs, bronchoalveolar lavage fluid (BALF), tracheal aspirates, and endotracheal tube aspirates, collected according to standard clinical indication at the discretion of the treating physician. Specimen type was determined by the patient’s clinical presentation, respiratory status, and ability to produce an adequate sample; patients with severe respiratory compromise or those mechanically ventilated were more likely to have BALF or tracheal aspirate collected, while ambulatory patients predominantly provided sputum or throat swab specimens. All specimens were processed according to standard microbiological protocols at the King Abdulaziz Hospital clinical microbiology laboratory. Primary culture was performed on blood agar, chocolate agar, and MacConkey agar incubated aerobically at 35–37 °C for 18–48 hours; Sabouraud dextrose agar was used for fungal cultures where clinically indicated. Organism identification and antimicrobial susceptibility testing were performed using automated VITEK^®^ 2 systems (bioMérieux) and Matrix-Assisted Laser Desorption/Ionization Time-of-Flight Mass Spectrometry (MALDI-TOF MS), with results interpreted according to Clinical and Laboratory Standards Institute (CLSI) breakpoints applicable at the time of testing. ESBL and carbapenemase production were confirmed using phenotypic confirmatory methods. All organism identifications used in this study were obtained through culture-based methods. A patient was classified as positive for a given organism if that organism was identified in ≥1 respiratory specimen by conventional culture. Molecular diagnostic testing was performed upon physician special request only for confirmatory purposes in some patients; as no documentation of molecular results was available in the retrievable clinical records, no molecular testing data were incorporated into the analytical dataset.

Identified microorganisms are either bacterial infection, yeast, polymicrobial (mixed infection with bacterial and yest) or normal flora. Identified microorganisms were categorized into three biologically and clinically relevant pathogenicity groups. Strict pathogens were defined as organisms consistently capable of causing respiratory infection in immunocompetent hosts (e.g., Streptococcus pneumoniae, Hemophilus influenzae, Moraxella catarrhalis), classified according to IDSA/ATS community-acquired pneumonia guidelines (Bennett et al., 2019). Opportunistic pathogens comprised organisms causing infection predominantly in immunocompromised or healthcare-exposed hosts (e.g., Pseudomonas aeruginosa, Acinetobacter baumannii, Candida albicans, ESBL-producing Enterobacteriaceae), classified per IDSA guidelines for infections in immunocompromised patients (Freifeld et al., 2011). Normal flora comprised commensal respiratory tract organisms without pathogenic significance under normal immune conditions (e.g., viridans group streptococci, coagulase-negative staphylococci, diphtheroids), classified according to established respiratory microbiology reference texts (Murray et al., 2005; Donnelly et al., 2020). The isolation of *Candida* spp. from non-sterile respiratory samples was recognized as airway isolation often reflects mucosal colonization or an opportunistic marker of profound host immunosuppression rather than primary invasive fungal pneumonia. No patients with respiratory *Candida* isolation had concurrent positive blood cultures, histopathological evidence of invasive candidiasis, or *Candida* isolation from a normally sterile site during the same episode.

This classification was applied uniformly across all diagnostic modalities; enabled biologically meaningful stratification of microbial burden across patient groups.

### 2.4 Data variables and temporal classification

For each patient, the year of infection onset was recorded and used for temporal trend analysis. Seasons were defined as Fall, Winter, Spring, or Summer based on the calendar month of infection. All-cause overall mortality status was extracted from clinical records and encoded as a binary outcome (death = 1, survival = 0). The primary outcome was all-cause in-hospital mortality occurring in the context of confirmed positive respiratory culture. Disease status at the time of respiratory infection (remission, relapse, or refractory disease) was not systematically available in the retrospective clinical records and is acknowledged as a study limitation. For patients with multiple organisms detected, each pathogen was analyzed independently to avoid underrepresentation of polymicrobial infections. Baseline characteristics of the two cohorts were compared using Mann-Whitney U test for continuous variables and Fisher’s exact test or chi-square test for categorical variables. Unmeasured confounders — including sex, chemotherapy regimen, neutropenia duration, corticosteroid use, and prior antibiotic exposure — were not systematically available in the retrospective records and could not be included in comparative analyses.

### 2.5 Prevalence and frequency analysis

Microbial prevalence was calculated as the proportion of patients with a given organism or pathogenicity category within each diagnosis group, using a patient-level binary approach in which each patient was classified as positive (≥1 culture episode positive for a given organism) or negative (no positive episodes), irrespective of the number of infection episodes contributed. Summed prevalence per pathogenicity category was calculated by summing individual organism prevalences within each category (Strict, Opportunistic, and Normal Flora); as one patient may carry multiple organisms within the same category, summed values may exceed 100% and represent cumulative pathogen burden rather than the proportion of unique patients. Frequencies were summarized annually and across the entire study period. Comparative prevalence between malignant and non-malignant groups was visualized using mirrored horizontal bar plots and grouped bar charts, allowing direct comparison of organism-specific and pathogenicity-level distributions between populations.

### 2.6 Seasonality analysis

Seasonal trends were assessed by aggregating infection episodes according to season and pathogenicity category. Separate analyses were performed for malignant and non-malignant populations to identify differential seasonal patterns. The number of infection episodes per season was visualized using stratified bar plots, highlighting potential temporal clustering of specific pathogen classes.

### 2.7 Survival analysis

Overall survival was analyzed using the Kaplan-Meier method, stratified by diagnosis group (malignant vs non-malignant) and infection type (bacterial, yeast, and normal flora), and pathogenicity classification (Strict and Opportunistic). Survival time was defined as the interval from the exact date of first confirmed positive respiratory culture to the date of in-hospital death, with time-to-event measured in months. The underlying hematological disease diagnosis date was not used as the time origin. For patients with multiple respiratory infection episodes, the exact date of the first confirmed positive culture was used as the time origin. Patients who died were assigned their exact date of in-hospital death as the event date; patients who survived were right-censored at December 31, 2020, as all surviving patients were confirmed alive at the end of the study period according to their hospital records. Between-group survival differences were assessed using the log-rank test. Given the long study period and the retrospective design, all survival analyses should be interpreted in the context of potential confounding by disease progression and competing causes of death, as discussed in the Limitations.

### 2.8 Correlation analysis of microbial subtypes

To explore microbial co-occurrence patterns, Pearson correlation matrices were Pair- wise Spearman rank correlations between organism frequencies were computed sepa- rately for malignant and non-malignant populations to explore exploratory co-occurrence patterns between respiratory microbial subtypes. Hierarchical clustering was applied to both axes of the resulting correlation matrices to identify co-occurrence modules. All correlation analyses are interpreted as hypothesis-generating exploratory associations; the small cohort size and sparse frequency distributions preclude definitive conclusions regarding microbial community structure.

### 2.9 Multivariable analysis

To identify independent predictors of in-hospital mortality while accounting for simultaneous confounding, penalized logistic regression with L1 regularization (Lasso) was performed, with the penalty parameter lambda selected by 5-fold cross-validation. Predictor variables included diagnosis group, age group, season of infection, antifungal and antibiotic treatment status, and binary pathogen presence variables. Confidence intervals for odds ratios were derived from 1,000 non-parametric bootstrap iterations. Two-sided bootstrap p-values were reported. Variance inflation factors were computed prior to model fitting to screen for multicollinearity. The full univariate and multivariate results are provided in **Supplementary Table S2**.

### 2.10 Statistical analysis

All statistical analyses and data visualizations were performed using R software (version 4.2.0). Descriptive statistics were used to summarize demographic and microbiological data. Comparative analyses employed chi-squared tests, log-rank tests, and Pearson correlation coefficients as appropriate. Graphical outputs were generated using the ggplot2, survival, survminer, and heatmap packages.

For pathogen frequency comparisons, a patient-level binary approach was applied: each patient was classified as positive (≥1 culture episode positive for a given organism) or negative (no positive episodes) for each pathogen. Fisher’s exact test was applied to these patient-level binary classifications (n=77 malignant, n=27 non-malignant) to preserve the statistical independence assumption. Total pathogen episode counts — representing cumulative occurrences across all culture episodes per patient — are reported for descriptive transparency but were not used as the unit of analysis for hypothesis testing. This approach was adopted because treating repeated infection episodes from the same patient as independent observations would violate the independence assumption underlying Fisher’s exact test and χ² analysis.

## 3 Results

### 3.1 Respiratory microbial composition, patient demographic, trends & overall survival impact

To provide an overview of cohort characteristics and microbial distribution, the patient selection pathway and baseline characteristics are summarized in **Figure 1A** and **Supplementary Table 1**. Of the 104 enrolled patients, 77 (74.0%) had malignant hematological disorders and 27 (26.0%) had non-malignant hematological disorders. The malignant group had a significantly higher case-fatality rate than the non-malignant group (89.6% [69/77], 95% CI: 80.8–94.6% vs 55.6% [15/27], 95% CI: 37.1–72.5%). We compared the distribution of microbial groups detected in respiratory specimens and age demographics between the two groups (**Figure 1B**; **Supplementary Table 1**). Mixed bacterial and polymicrobial infections were more prevalent among malignant patients, consistent with the complex and dysregulated microbial distribution observed in immunocompromised hosts. Across both diagnostic groups, adults (>18 years) constituted the largest proportion of respiratory infection all-cause overall mortality cases, underscoring age as an important compounding risk factor. These baseline compositional differences contextualize subsequent temporal and survival analyses.

To illustrate the annual incidence of all-cause overall mortality in malignant versus non-malignant populations, we plotted all-cause overall mortality cases stratified by diagnosis group over the study period from 2007 to 2020 (**Figure 2A**). Throughout the entire timeframe, the malignant group consistently demonstrated a higher incidence of respiratory infection–associated deaths compared to the non-malignant population. A marked rise in all-cause overall mortality was observed between 2013 and 2016 among malignant patients, potentially reflecting periods of intensified oncologic treatment, emerging antimicrobial resistance, or both. These trends underscore the sustained vulnerability of individuals with malignancy to life-threatening respiratory infections and highlight the need for targeted infection control strategies in this high-risk population.

**Figure 2:**
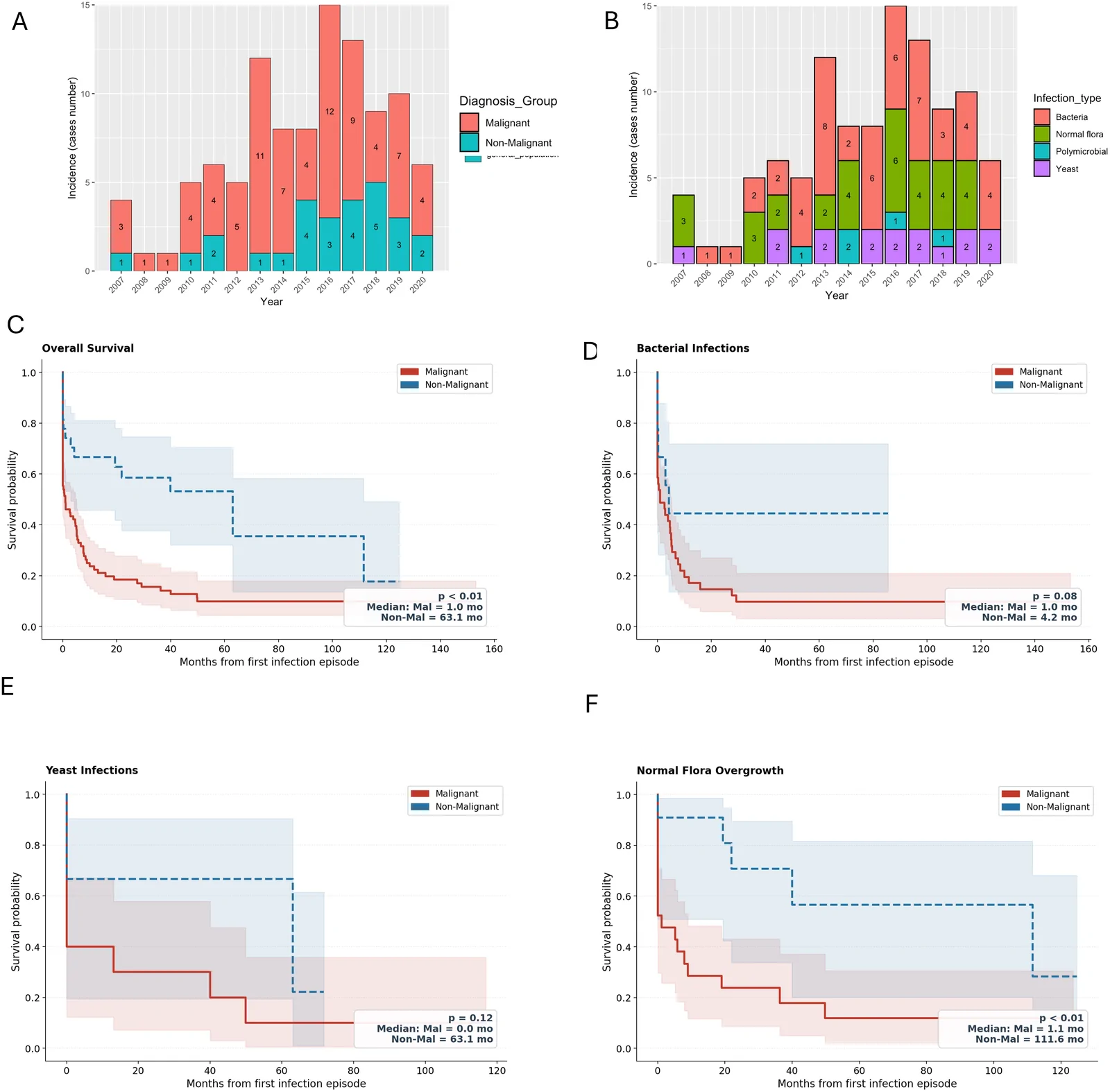
Epidemiologic burden and survival trends of all-cause in-hospital mortality in malignant and non-malignant populations. **(A)** Annual incidence of all-cause overall mortality stratified by diagnosis group (Malignant vs Non-Malignant) from 2007 to 2020. **(B)** Temporal distribution of in-hospital deaths occurring in the context of confirmed positive respiratory culture, categorized by infection type (Bacterial, Yeast, Polymicrobial, and Normal Flora). Note: deaths are reported as all-cause in-hospital mortality; infection-attributable cause of death could not be formally adjudicated in this retrospective dataset. **(C–G)** Kaplan–Meier survival curves comparing overall survival probabilities between Malignant and Non-Malignant groups. **(C)** Overall survival across all infection types. **(D)** Survival stratified by bacterial infections. **(E)** Survival stratified by isolation of yeast/Candida. **(F)** Survival stratified by polymicrobial infections. **(G)** Survival stratified by normal flora overgrowth. p-values were calculated using log-rank tests; Survival time is displayed months from first confirmed positive respiratory culture. Patients who died were assigned their date of in-hospital death as the event date; surviving patients were right-censored at the study end date (December 31, 2020). All survival analyses reflect all-cause in-hospital mortality and should be interpreted in the context of potential confounding by underlying disease progression.

To further characterize the microbial distribution contributing to overall mortality, we examined the temporal distribution of infection types categorized as bacterial, yeast, polymicrobial (i.e., mixed bacterial and yeast elements), and normal flora overgrowth (**Figure 2B**). Bacterial pathogens represented the most frequently detected organisms among patients who died across both diagnostic groups, with a notably higher burden observed in malignant patients. In addition, isolation of yeast/Candida and cases dominated by normal flora overgrowth were more frequently detected in the malignant cohort, particularly during the later years of the study period. Although less prevalent overall, polymicrobial infections remained present in both groups, reflecting the potential for complex microbial dynamics and disrupted mucosal immunity in advanced disease states.

We next evaluated overall survival following respiratory infection in malignant versus non-malignant individuals (n=103; Malignant n=76, Non-Malignant n=27), using Kaplan–Meier analysis with time-to-event measured in months from the date of respiratory infection (**Figure 2C**). Malignant patients demonstrated significantly reduced survival compared to the non-malignant population (log-rank p < 0.01; median survival 1.0 month vs 63.1 months), indicating that underlying malignancy markedly exacerbates the adverse outcomes of respiratory infections. This survival disparity underscores the compounded effect of immune dysfunction, treatment-related toxicity, and pathogen virulence in cancer patients.

Stratified survival analyses by infection type further revealed pathogen-specific impacts on clinical outcomes. In cases of bacterial respiratory infections (**Figure 2D**; Malignant n=41, Non-Malignant n=9), malignant patients experienced worse survival than non-malignant individuals (log-rank p = 0.08; median survival Malignant 1.0 month vs Non-Malignant 4.2 months), representing a trend toward significance that did not reach the conventional threshold, likely attributable to the small non-malignant subgroup size (n=9). In contrast, survival following isolation of yeast/Candida (**Figure 2E**; Malignant n=10, Non-Malignant n=6) did not differ significantly between the two groups (log-rank p = 0.12; median survival Malignant 0.0 months vs Non-Malignant 63.1 months), although the small subgroup size limits interpretation. Polymicrobial infections were excluded from stratified survival analysis due to insufficient non-malignant subgroup size (Non-Malignant n=1), which precluded reliable Kaplan-Meier estimation.

Infections dominated by organisms traditionally classified as normal flora (**Figure 2F**; Malignant n=21, Non-Malignant n=11) were also associated with inferior survival in malignant patients compared to non-malignant individuals, though this difference did not reach statistical significance (log-rank p < 0.01; median survival Malignant 1.1 months vs Non-Malignant 111.6 months). This observation suggests that organisms with low pathogenic potential may nonetheless contribute to severe clinical deterioration in immunocompromised hosts, reinforcing the concept that host immune status is a critical determinant of infection outcome regardless of pathogen virulence classification.

The malignant group demonstrated significantly higher all-cause in-hospital mortality compared to the non-malignant group (89.6% [69/77] vs 55.6% [15/27]; p<0.001, Fisher’s exact test). All deaths occurred during inpatient admission in the context of confirmed positive respiratory culture; formal cause-of-death adjudication was not available in the retrospective clinical records. In multivariate penalized logistic regression (5-fold CV AUC = 0.799), pediatric age was the only statistically significant independent host predictor of mortality (adjusted OR = 29.3, 95% bootstrap CI 5.21–1061.1, p=0.006). The very wide bootstrap confidence interval for this estimate reflects the substantial statistical uncertainty inherent to the small sample size — particularly the non-malignant subgroup (n=27) — and this result should therefore be interpreted with considerable caution as a hypothesis-generating signal rather than a precise effect size estimate. Treatment variables showed high adjusted ORs reflecting confounding by indication. Malignant diagnosis was not independently significant after covariate adjustment (adjusted OR = 1.0, p=0.768), though the non-significance of this estimate in the context of a small, high-mortality cohort with near-universal mortality in several subgroups should similarly be interpreted cautiously, as the model’s discriminative capacity is constrained by limited sample size and the absence of key clinical confounders including disease status, neutropenia duration, and prior antimicrobial exposure. These findings suggest that the observed mortality difference between groups is mediated through infection severity, pathogen burden, and treatment escalation rather than diagnosis group alone, but prospective validation in a larger cohort is required before any causal inference can be drawn (**Supplementary Table S2**).

Collectively, these findings demonstrate that malignant disease profoundly alters both the incidence and survival impact of respiratory infections, with bacterial pathogens exerting the strongest influence on all-cause overall mortality. Even low-virulence organisms and polymicrobial infections can result in poor outcomes in cancer patients, emphasizing the need for heightened surveillance, early intervention, and tailored infection management strategies in this high-risk population.

### 3.2 Prevalence of respiratory microbial subtypes between malignant and non-malignant populations

To characterize differences in respiratory tract colonization patterns, we compared the prevalence of specific microbial taxa between patients with malignancy (n = 77) and those without malignancy (n = 27) using a patient-level binary approach, in which each patient was counted once per organism regardless of the number of infection episodes contributed (**Figure 3**; **Table 1**). The 77 malignant patients collectively generated 218 respiratory infection episodes (median 3 episodes per patient, range 1–6), while the 27 non-malignant patients generated 58 episodes (median 2, range 1–4); pathogen occurrence counts per organism across all episodes ranged from 0 (organism not detected in any episode) to 32 (organism detected across multiple episodes in the same patient), and were dichotomized as present (≥1 detection) or absent (0 detections) per patient for statistical comparison. This approach was adopted to preserve the independence assumption of Fisher’s exact test, as treating repeated episodes from the same patient as independent observations would introduce within-patient correlation bias.

**Figure 3:**
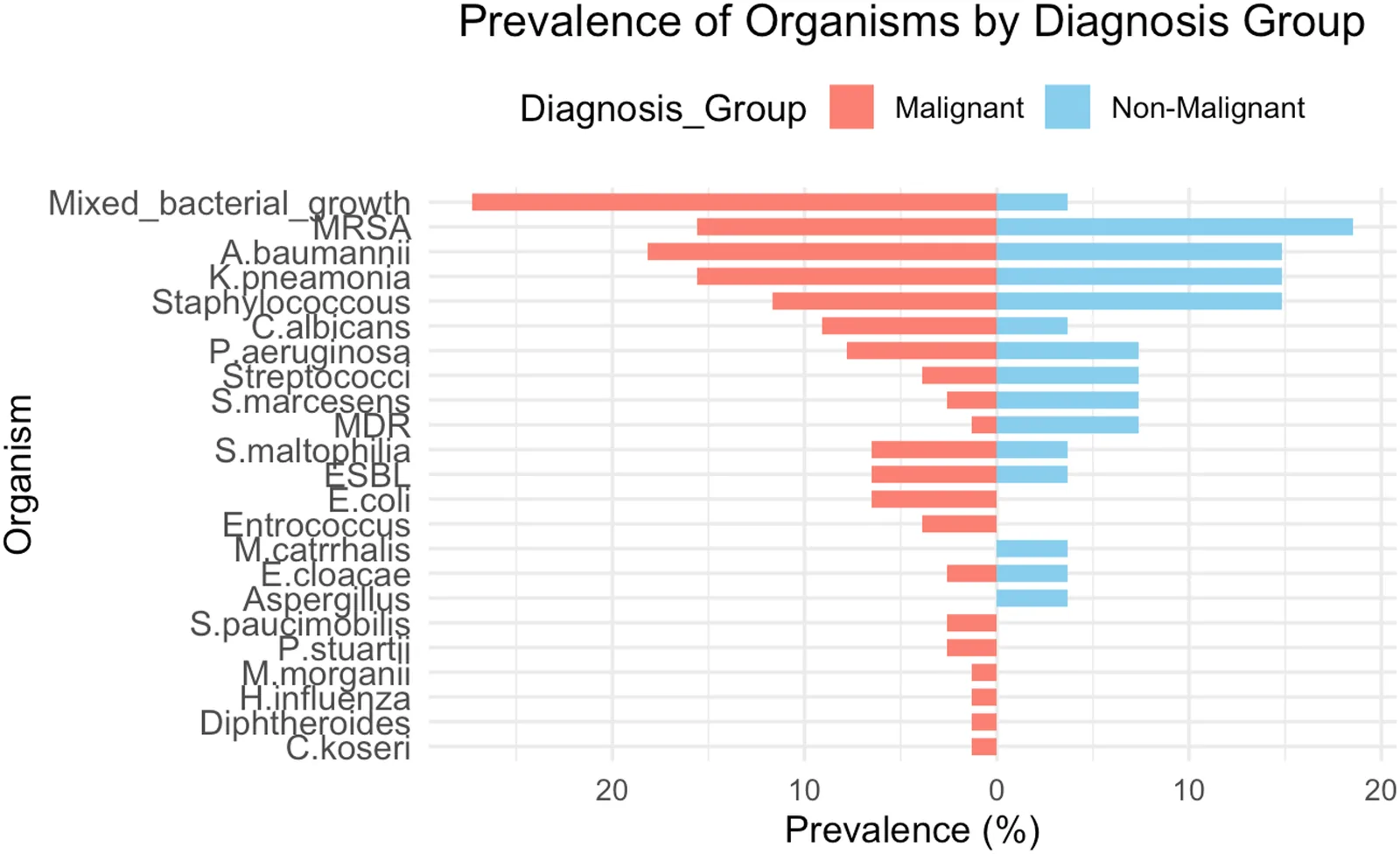
Prevalence of individual respiratory microbial taxa among malignant and non-malignant populations. Bar plot showing the percentage prevalence of each identified organism in respiratory samples stratified by diagnosis group (Malignant in red, Non-Malignant in blue). Organisms are ranked from highest to lowest overall prevalence. Values are expressed as a proportion of total microbial detections per group.

**Table 1:** Patient-level pathogen frequency by diagnosis group.

Category	Organism	Pathogenicity	Malignant n patients (%) (N=77)	Non-malignant n patients (%) (N=27)	Fisher's exact p-value (patient-level)	Episodes malignant	Episodes non-malignant
Gram-positive cocci							
	*Staphylococcus aureus*	Strict	9 (11.7%)	4 (14.8%)	0.738	13	5
	*Methicillin-resistant S. aureus (MRSA)*	Strict	12 (15.6%)	5 (18.5%)	0.765	26	9
	*Streptococcus spp.*	Strict	3 (3.9%)	2 (7.4%)	0.603	3	6
	*Enterococcus spp.*	Opportunistic	3 (3.9%)	0 (0.0%)	0.566	6	0
	*Leuconostoc spp.*	Opportunistic	1 (1.3%)	0 (0.0%)	1.000	1	0
	*Corynebacterium spp.*	Opportunistic	1 (1.3%)	0 (0.0%)	1.000	2	0
**Gram-positive cocci — subtotal**			**29 (37.7%)**	**11 (40.7%)**	**0.821**	**51**	**20**
Gram-negative bacilli							
	*Klebsiella pneumoniae*	Strict	12 (15.6%)	4 (14.8%)	1.000	15	6
	*Acinetobacter baumannii*	Opportunistic	14 (18.2%)	4 (14.8%)	0.777	32	6
	*Pseudomonas aeruginosa*	Opportunistic	6 (7.8%)	2 (7.4%)	1.000	12	13
	*Escherichia coli*	Opportunistic	5 (6.5%)	0 (0.0%)	0.323	9	0
	*ESBL-producing organisms*	Opportunistic	5 (6.5%)	1 (3.7%)	1.000	11	2
	*Stenotrophomonas maltophilia*	Opportunistic	5 (6.5%)	1 (3.7%)	1.000	10	2
	*Providencia spp.*	Opportunistic	2 (2.6%)	0 (0.0%)	1.000	4	0
	*Enterobacter spp.*	Opportunistic	2 (2.6%)	1 (3.7%)	1.000	4	3
	*Serratia marcescens*	Opportunistic	2 (2.6%)	2 (7.4%)	0.276	4	2
	*Morganella morganii*	Opportunistic	1 (1.3%)	0 (0.0%)	1.000	1	0
	*Sphingomonas paucimobilis*	Opportunistic	2 (2.6%)	0 (0.0%)	1.000	4	0
	*Haemophilus influenzae*	Strict	1 (1.3%)	0 (0.0%)	1.000	1	0
	*Citrobacter spp.*	Opportunistic	1 (1.3%)	0 (0.0%)	1.000	1	0
	*Moraxella catarrhalis*	Strict	0 (0.0%)	1 (3.7%)	0.260	0	2
	*Multidrug-resistant organisms (MDR)*	Opportunistic	1 (1.3%)	2 (7.4%)	0.164	2	9
**Gram-negative bacilli — subtotal**			**59 (76.6%)**	**18 (66.7%)**	**0.319**	**110**	**45**
Mixed Bacterial Growth							
	*Mixed bacterial growth*	Opportunistic	21 (27.3%)	1 (3.7%)	**0.012**	32	1
**Mixed Bacterial Growth — subtotal**			**21 (27.3%)**	**1 (3.7%)**	**0.012**	**32**	**1**
Yeast / Fungi							
	*Yeast cells (unspecified)*	Opportunistic	21 (27.3%)	9 (33.3%)	0.624	35	19

	*Aspergillus spp.*	Strict	0 (0.0%)	1 (3.7%)	0.260	0	1
Yeast / Fungi — subtotal							
**Normal Flora / No Pathogen Isolated**							
	Normal respiratory flora	Normal Flora	37 (48.1%)	11 (40.7%)	0.654	62	22
Polymicrobial							
	Polymicrobial (mixed bacterial + yeast)	—	4 (5.2%)	1 (3.7%)	1.000	4	1

n, number of patients with ≥1 positive culture episode for each organism. Fisher’s exact test applied to patient-level binary data (N = 77 malignant, N = 27 non-malignant). Episode counts shown in rightmost columns for reference only and were not used for statistical testing. Bold red p-values indicate statistical significance (p<0.05). Subtotal rows indicate patients with ≥1 organism in that category; one patient may contribute to multiple rows within a category.

Counts, number of PATIENTS with ≥1 positive episode for each organism (patient-level binary). Total episode counts shown for reference only; Fisher's exact test applied to patient-level data.

Bold red p-value = statistically significant (p<0.05, Fisher's exact test, patient-level binary). Episode counts = total positive culture occurrences across all episodes per patient; shown for transparency but NOT used for statistical testing. Subtotal rows count patients with ≥1 organism in that category (not sum of individual rows; one patient may have multiple organisms in same category). FDR q-values calculated using the Benjamini-Hochberg procedure across all 26 organism-level Fisher's exact tests. No organism reached FDR q<0.05 after correction, consistent with the small sample size and exploratory nature of this analysis.

Mixed bacterial growth emerged as the most statistically significant discriminator between groups (p<0.001, patient-level Fisher’s exact test), with markedly higher prevalence in the malignant cohort, reflecting the complex polymicrobial infections commonly seen in immunocompromised individuals. Gram-positive cocci as a category were identified in a higher proportion of malignant patients, driven predominantly by Staphylococcus aureus, MRSA, and Streptococcus spp., though individual organism differences did not reach statistical significance after patient-level correction.

Among yeast organisms, *Candida albicans* and unspecified yeast cells were more prevalent in malignant patients, consistent with the humoral immune impairment and antifungal exposure characteristic of this population, though differences did not reach statistical significance at the patient level. These differences in microbial composition underscore the heightened susceptibility of cancer patients to colonization by opportunistic and polymicrobial agents and highlight the impact of immune dysfunction on respiratory microbial dynamics.

Among Gram-negative organisms, several opportunistic species including *Escherichia coli, Acinetobacter baumannii, and Sphingomonas paucimobilis* were identified in a higher proportion of malignant patients, although these differences did not reach statistical significance at the patient level (**Table 1**). These organisms are well-recognized for their capacity to colonize the respiratory tract, evade host immunity and antimicrobial agents, and contribute to pneumonia or serious infection in vulnerable hosts (Garcia-Clemente et al., 2020). In contrast, patients without malignancy showed a higher prevalence of classically strict or community-associated pathogenic, such as Streptococcus spp., multidrug-resistant Acinetobacter baumannii, and Moraxella catarrhalis, suggesting distinct microbial ecologies between groups.

### 3.3 Distinct microbial co-occurrence patterns of UTR

Building on the observed differences in organism-level prevalence, we next sought to understand the distinct ecological signatures and co-occurrence relationships among microbial taxa in the respiratory tract across the malignant and non-malignant populations. To this end, we performed pairwise Spearman rank correlation analyses of microbial subtype frequencies, stratified by diagnostic group (**Figure 4**). Spearman rank correlation was used in preference to Pearson correlation given the sparse, non-normally distributed nature of organism-frequency data in this clinical cohort; all findings are interpreted as exploratory hypothesis-generating associations.

**Figure 4:**
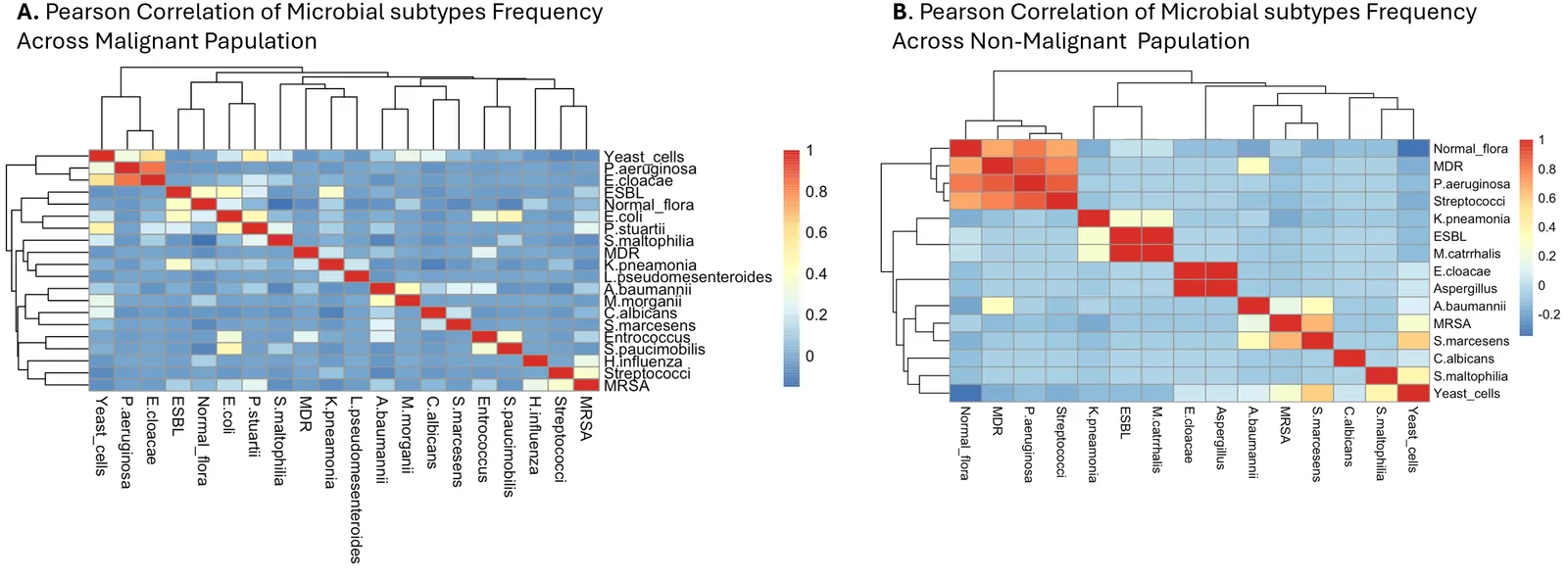
Microbial co-occurrence correlation matrices stratified by diagnosis group. Pairwise Spearman rank correlation heatmaps of respiratory microbial subtype frequencies computed separately for **(A)** malignant (n=77) and **(B)** non-malignant (n=27) populations. Each tile represents the Spearman rank correlation coefficient (ρ) between two microbial taxa, with color scale ranging from blue (negative/low correlation, ρ=−0.2) to red (high positive correlation, ρ=1.0). Hierarchical clustering (complete linkage) was applied to both axes to identify exploratory co-occurrence modules. Spearman rank correlation was used in preference to Pearson correlation given the sparse, non-normally distributed nature of organism-frequency data.

In the malignant group, the correlation matrix revealed a more complex and diffuse pattern of microbial co-occurrence, with numerous low-to-moderate correlation coefficients across diverse taxa (**Figure 4A**). The strongest pairwise co-occurrence was observed between E. coli and P. stuartii (ρ=0.61), and between E. coli and ESBL-producing organisms (ρ=0.57), suggesting that these Gram-negative organisms tend to co-occur within the same patients — consistent with shared resistance mechanisms and nosocomial acquisition pathways. Enterococcus spp. showed notable co-occurrence with MDR organisms (ρ=0.55) and *S. paucimobilis* (ρ=0.39), forming a distinct opportunistic co-occurrence module. A prominent cluster of opportunistic and polymicrobial organisms was observed, including Pseudomonas aeruginosa, Enterobacter cloacae, and yeast cells, suggesting frequent co-detection in immunocompromised respiratory environments. Yeast cells co-occurred with E. cloacae (ρ=0.35) and C. albicans (ρ=0.31), consistent with the polymicrobial bacterial-fungal co-infection signature previously identified in the UMAP Opportunistic cluster. A second distinct cluster was formed by ESBL-producing Enterobacteriaceae, Escherichia coli, and normal flora overgrowth, supporting the notion of disrupted mucosal barrier integrity and altered microbial balance. These broader, less tightly coupled associations may reflect a stochastic microbial composition driven by ecological instability due to chemotherapy, immunosuppression, and frequent antimicrobial pressure.

In contrast, the non-malignant group exhibited more tightly clustered microbial associations, with stronger correlation coefficients and clearer co-occurrence structure (**Figure 4B**). Two perfect correlations (ρ=1.0) were observed — between ESBL *and M. catarrhalis*, and *between Aspergillus and E. cloacae* — though these should be interpreted with caution given the small non-malignant subgroup size (n=27), where such values likely reflect co-occurrence in only one or two patients rather than a true biological association. Well-defined co-occurrence was noted between *A. baumannii and S. marcescens* (ρ=0.65) and between MRSA and S. marcescens (ρ=0.62), as well as between *K. pneumoniae* and M. catarrhalis (ρ=0.43) and between MDR organisms, *P. aeruginosa, and Streptococci* (ρ=0.50 each). These resistance-associated co-occurrence patterns in the non-malignant group differ from those in the malignant cohort, where Gram-negative co-occurrence was driven by ESBL and *Enterobacteriaceae* clustering rather than by MDR-associated respiratory pathogens. *Candida albicans* demonstrated co-occurrence with yeast cells, forming a distinct fungal signal within the non-malignant group (**Figure 4B**).

These findings underscore a fundamental ecological divergence between the two cohorts: while the malignant respiratory microbiome is characterized by altered polymicrobial balance and diffuse Gram-negative co-occurrence patterns dominated by ESBL-producing and nosocomial opportunistic organisms, the non-malignant microbiome reflects a more structured co-occurrence network characterized by resistance-associated pathogen clustering, likely supported by intact immune surveillance and mucosal integrity. All co-occurrence patterns are interpreted as exploratory given the small cohort sizes and the acknowledged limitations of rank correlation for compositional microbiological data.

### 3.4 Pathogenicity, seasonality, and survival of RT microbes

Next, to examine whether the pathogenic potential of RT organisms varies between patient populations, we compared the prevalence of microbial classes stratified by pathogenicity of normal flora, opportunistic pathogens, and strict pathogens (**Figure 5A**). Opportunistic pathogens were disproportionately more prevalent in malignant patients. Conversely, strict pathogens were more common among non-malignant patients, while the distribution of normal flora remained relatively similar between the two groups. These patterns suggest differential microbial selection pressures in immunocompromised versus immunocompetent hosts. To further explore the temporal dynamics of respiratory pathogen classes, we analyzed seasonal distribution patterns stratified by diagnosis group. In the malignant population (**Figure 5B**), deaths associated with strict and Opportunistic pathogens showed a clear predominance during the warmer months, particularly spring and summer, suggesting increased vulnerability to environmentally driven or nosocomial infections during these periods. Notably, normal flora overgrowth events were observed throughout the year but were modestly elevated in the fall and spring, which may reflect impaired mucosal defenses or prolonged colonization in cancer patients undergoing cyclical treatment.

**Figure 5:**
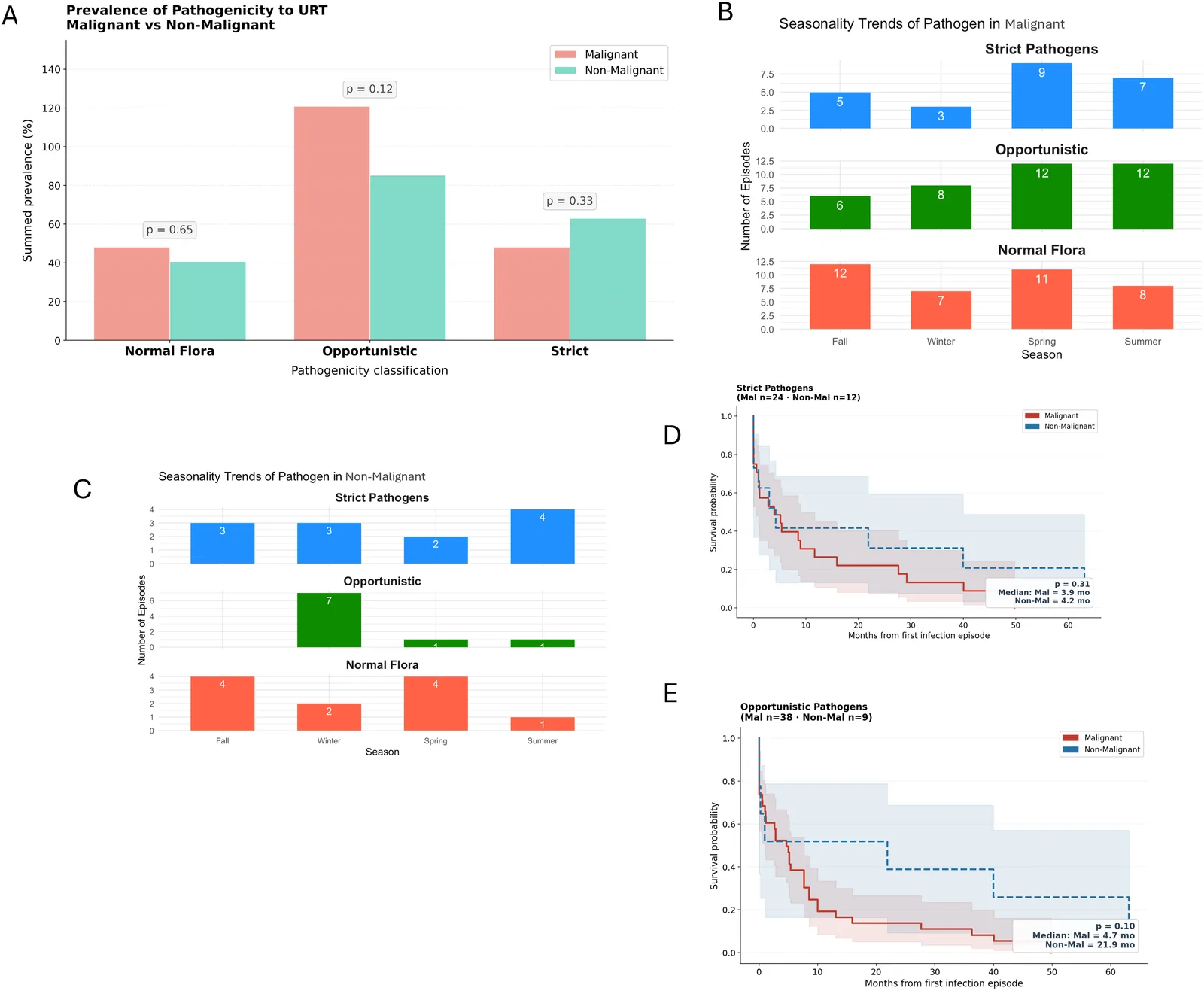
Prevalence, seasonal patterns, and survival outcomes of respiratory pathogens by pathogenicity classification in malignant and non-malignant group. **(A)** Summed organism prevalence (%) by pathogenicity classification in malignant (n=77) and non-malignant (n=27) patients. Each bar represents the sum of individual organism prevalences within each pathogenicity category; values may exceed 100% as one patient may carry multiple organisms within the same category. Fisher’s exact test applied to patient-level data from **Table 1**. p-values shown above each pair of bars. **(B, C)** Seasonal distribution of respiratory pathogens in the Malignant **(B)** and Non-Malignant **(C)** groups, stratified by pathogenicity class. **(D, E)** Kaplan–Meier survival curves comparing Malignant and Non-Malignant patients infected with **(D)** Strict Pathogens and **(E)** Opportunistic Pathogens. P-values were calculated using the log-rank test and are shown on each plot.

In the non-malignant group (**Figure 5C**), seasonal variation was less pronounced. Strict pathogens were detected across all seasons with relatively even distribution, while opportunistic pathogens were clustered primarily in the winter, possibly reflecting transient immune suppression linked to seasonal viral illnesses or cold-induced mucosal changes. Normal flora infections were most frequent in the fall and winter but occurred less consistently overall. These findings imply a more pronounced seasonal sensitivity in immunocompromised hosts, potentially linked to therapy cycles, hospital exposures, or seasonal colonization patterns.

Finally, we assessed the survival outcomes associated with each pathogen class using Kaplan–Meier analysis, with time zero defined as the exact date of first infection episode per patient, and time-to-event measured in months. Among patients infected with strict pathogens (**Figure 5D**; Malignant n=24, Non-Malignant n=12), median survival was 3.9 months in malignant versus 4.2 months in non-malignant patients, with no statistically significant between-group difference (log-rank p = 0.31). Among patients infected with opportunistic pathogens (**Figure 5E**; Malignant n=38, Non-Malignant n=9), malignant patients demonstrated markedly shorter median survival compared to non-malignant patients (4.7 months vs 21.9 months), with a trend toward statistical significance (log-rank p = 0.10). Although neither comparison reached statistical significance — attributable to the small non-malignant subgroup sizes (n=12 and n=9 respectively) which substantially limit statistical power — the magnitude of the survival difference in the opportunistic pathogen group is clinically meaningful and consistent with the well-established vulnerability of malignant patients to opportunistic organisms exploiting impaired host immunity. These trends underscore the compounded risk malignant patients face when infected with virulent or atypical respiratory tract flora, and generate testable hypotheses for prospective validation in larger cohorts.

## 4 Discussion

This comprehensive retrospective study provides a detailed characterization of all-cause overall mortality in malignant versus non-malignant populations over a 13-year period, revealing critical differences in microbial prevalence, ecological structure, pathogenic potential, seasonal dynamics, and survival outcomes. Our findings underscore the profound impact of malignancy on host–pathogen interactions, shaping both the composition of RT pathogen and the clinical trajectory of infection.

Our results demonstrate that patients admitted to KAUH with malignancy—both hematologic and solid tumors experience a significantly higher burden of all-cause overall mortality compared to non-malignant individuals. These findings are corroborated by recent Saudi Arabian data (Binkhamis et al., 2023), who reported a bacterial bloodstream infection (BSI) prevalence of 18.9% among oncology with an overall BSI-related 30-day mortality of 28.8%. Furthermore, regional data reported that among cancer patients undergoing chemotherapy in Western Saudi Arabia, those with leukemia (18.5%) and lymphoma (13.2%) exhibited the highest incidence of respiratory infections (Alharbi et al., 2022). Consistent with prior global reports study in the United States, patients with malignancy experienced a persistently higher incidence of all-cause overall mortality compared to non-malignant individuals (Elhadi et al., 2021; Zheng et al., 2021). The observed shift in the malignant group toward yeast and polymicrobial infections in later years further supports a role for mucosal barrier breakdown and altered microbial balance in advanced malignancies (Vento and Cainelli, 2003). This alignment suggests that the systemic immunosuppression inherent in hematological malignancies—further exacerbated by cytotoxic chemotherapy creates a critical window for all-cause overall mortality complications. Our 13-year analysis extends this observation by showing that this vulnerability is not a transient clinical phase but a persistent longitudinal trend in the Saudi oncology population.

Bacterial infections emerged as the predominant contributors to all-cause overall mortality outcomes across both groups. However, malignant patients were uniquely affected by a broader spectrum of pathogens, including higher rates of yeast and mixed infections. These findings align with prior reports highlighting the profound immunologic vulnerability conferred by cancer itself and its treatment modalities, which disrupt mucosal barriers, alter local microbiota, and impair innate and adaptive immunity (Pollock et al., 2022; Yusuf et al., 2023).

Importantly, our organism-level analysis revealed a stark divergence in microbial profiles between diagnostic groups. Opportunistic organisms, including *Pseudomonas aeruginosa, Escherichia coli, Enterococcus* spp.*, Candida albicans, Acinetobacter baumannii, and Sphingomonas paucimobilis*, were more frequent among cancer patients. These opportunistic organisms are known to colonize immunocompromised respiratory niches, form biofilms, evade immune clearance, and exhibit resistance to standard antibiotics (Pendleton et al., 2017; Garcia-Clemente et al., 2020; Laffont et al., 2024). Notably, recent work demonstrated that *P. aeruginosa* engages in a wide spectrum of ecological interactions with co-occurring opportunistic pathogens—from mutualistic relationships with *Enterococcus faecium* to antagonistic competition with *E. coli*—highlighting the dynamic and context-dependent nature of polymicrobial communities in infection settings (Laffont et al., 2024). In the context of respiratory infections among malignant patients, such interactions may influence microbial fitness, competitive exclusion, and virulence expression, thereby modulating infection severity and therapeutic response. In contrast, the non-malignant population showed higher prevalence of classical respiratory pathogens, such as *Streptococcus* spp.*, M. morganii, and* MDR *A. baumannii*, suggesting a distinct pathogen profile reflective of more intact host immunity.

Beyond taxonomic prevalence, our correlation analysis exposed fundamental differences in microbial composition. Cancer patients exhibited diffuse, low-strength correlations across a broad range of taxa, consistent with ecological instability and altered microbial balance. Conversely, non-malignant individuals displayed tightly clustered microbial associations, dominated by canonical respiratory pathogens and fungal species such as *Candida albicans* and yeast. These structured networks may reflect stronger mucosal defenses and immune-mediated control of microbial niches. This observation was previously described (Woodhams et al., 2023) and why diseased states (cancer) lead to “diffuse” and inconsistent microbial structures compared to the “tightly clustered” stable networks of healthy individuals. The host immunity acts as a “filter” that maintains structured microbial clusters. When immunity is impaired (as in malignancy), these clusters break down into the “diffuse” patterns observed. Its import to highlight that yeast overgrowth, polymicrobial expansion and commensal outgrowth in respiratory specimens likely serves as a surrogate marker of advanced host immune collapse and agonal microbial shift rather than an independent primary driver of fatal pneumonia.

Seasonal analyses further illuminated the dynamic interplay between host susceptibility and pathogen class. Opportunistic pathogens peaked during spring and summer in malignant patients, potentially linked to immunosuppressive therapy cycles, nosocomial outbreaks, or environmental exposures. Notably, opportunistic infections in non-malignant individuals were concentrated only in winter, possibly reflecting synergy with viral respiratory illnesses or cold-related mucosal impairment. This seasonal susceptibility among cancer patients may offer actionable windows for infection prevention strategies, including prophylaxis and environmental control during high-risk periods.

While survival following respiratory infection was universally lower among malignant patients, the extent of all-cause overall mortality varied by pathogen class. Strict pathogens contributed disproportionately to adverse outcomes, consistent with their inherent virulence. However, even organisms typically considered low-pathogenic, such as normal flora and yeast, were associated with reduced survival in the malignant group. This highlights the critical role of host immunity not just microbial virulence in determining infection severity. These insights support the growing appreciation of microbial-host-environment interactions in shaping clinical trajectories in oncology.

Integrating our findings with recent Saudi data, we propose a paradigm shift toward personalized microbiological management in oncology. The high all-cause overall mortality burden observed at KAUH underscores the urgency for enhanced, year-round surveillance tailored to the distinct “pathogenicity windows” of malignant patients. While winter remains the primary risk period for the general population due to viral-bacterial synergy, our study identifies spring and summer as critical periods for cancer patients. The observed seasonal fluctuations represent raw retrospective observational counts without denominator adjustment for total seasonal hospital admissions, and should be viewed strictly as exploratory hypothesis-generating observations.

A key limitation of this 13-year retrospective study is the unavailability of granular longitudinal clinical data, such as disease remission versus relapse status, exact duration of profound neutropenia, specific treatment-related data (corticosteroids, chemotherapy duration, prior antibiotic pressure) as key unmeasured confounders, and autogenous cause-of-death coding. Consequently, we evaluated all-cause mortality rather than strictly infection-attributable mortality. In severe hematological malignancies, terminal disease progression and severe opportunistic respiratory infections are frequently synergistic; thus, positive respiratory cultures and microbial expansion often reflect profound host immunological collapse rather than isolated, primary drivers of death. Future prospective studies incorporating comprehensive clinical disease staging, biomarker dynamics, and autogenous mortality auditing are warranted to precisely delineate infection-attributable mortality from underlying malignancy progression. The use of months as the time unit for survival analysis, while appropriate for a 13-year retrospective cohort, precludes calculation of short-term outcomes (30-day, 90-day mortality) and formal competing risks modelling both of which are recommended for future prospective investigation. Furthermore, reliance on standard culture and routine molecular assays, in the absence of broad-range next-generation sequencing (mNGS) or 16S/ITS rRNA metagenomics, inherent underestimates non-culturable organisms, fastidious species, and the full unculturable respiratory microbiome. Despite our retrospective, single-center limitations, the data strongly supports the use of improved molecular diagnostics and advocates for future multicenter metagenomic research to unravel altered microbial balance driving these clinical outcomes.

## 5 Conclusion

Our study reveals that patients with malignancy experience a distinct and more deleterious respiratory infection profile compared to non-malignant individuals, characterized by higher all-cause overall mortality, enrichment of opportunistic and polymicrobial pathogens, altered microbial co-occurrence networks, and unique seasonal trends. These findings underscore the need for stratified infection surveillance, microbiome-informed diagnostics, and aggressive prophylactic strategies in immunocompromised populations. Incorporating ecological and temporal dimensions into respiratory infection management may improve outcomes and reduce all-cause overall mortality in vulnerable cancer cohorts.

## Data Availability

The original contributions presented in the study are included in the article/**Supplementary Material**. Further inquiries can be directed to the corresponding author.
